# Optimal treatment strategy and prognostic analysis for patients with non-metastatic pT4 colon adenocarcinoma

**DOI:** 10.3389/fonc.2023.1342289

**Published:** 2024-01-08

**Authors:** Xinyue Zhao, Qinghong Meng, Mengyun Zhou, Judong Luo, Lijun Hu

**Affiliations:** ^1^ Graduate School of Dalian Medical University, Dalian, China; ^2^ Department of Radiation Oncology, Changzhou No. 2 People’s Hospital Affiliated to Nanjing Medical University, Changzhou, China

**Keywords:** pT4M0 colon cancer, adenocarcinoma, prognosis, model, treatment modality

## Abstract

**Objective:**

This study endeavored to explore the optimal treatment strategy and conduct a prognostic analysis for patients diagnosed with pT4M0 (pathologic stage T4) colon adenocarcinoma (COAD).

**Methods and materials:**

A total of 8,843 patients diagnosed with pT4M0 COAD between January 2010 and December 2015 were included in this study from the Surveillance, Epidemiology, and End Results (SEER) database. These patients were randomly divided into a training set and an internal validation set using a 7:3 ratio. Variables that demonstrated statistical significance (P<0.05) in univariate COX regression analysis or held clinical significance were incorporated into the multivariate COX regression model. Subsequently, this model was utilized to formulate a nomogram. The predictive accuracy and discriminability of the nomogram were assessed using the C-index, area under the curve (AUC), and calibration curves. Decision curve analysis (DCA) was conducted to confirm the clinical validity of the model.

**Results:**

In the entire SEER cohort, the 3-year overall survival (OS) rate (74.22% vs. 63.20%, P<0.001) and the 3-year cancer-specific survival (CSS) rate (76.25% vs. 66.98%, P<0.001) in the surgery combined with postoperative adjuvant therapy (S+ADT) group surpassed those in the surgery (S) group. Multivariate COX regression analysis of the training set unveiled correlations between age, race, N stage, serum CEA (carcinoembryonic antigen), differentiation, number of resected lymph nodes, and treatment modalities with OS and CSS. Nomograms for OS and CSS were meticulously crafted based on these variables, achieving C-indexes of 0.692 and 0.690 in the training set, respectively. The robust predictive ability of the nomogram was further affirmed through receiver operating characteristic (ROC) and calibration curves in both the training and validation sets.

**Conclusion:**

In individuals diagnosed with pT4M0 COAD, the integration of surgery with adjuvant chemoradiotherapy demonstrated a substantial extension of long-term survival. The nomogram, which incorporated key factors such as age, race, differentiation, N stage, serum CEA level, tumor size, and the number of resected lymph nodes, stood as a dependable tool for predicting OS and CSS rates. This predictive model held promise in aiding clinicians by identifying high-risk patients and facilitating the development of personalized treatment plans.

## 1 Introduction

Colon adenocarcinoma (COAD) ranks among the most prevalent malignant tumors of the digestive system. Global statistics from 2018 reveal an alarming incidence, with over 1.8 million new cases of colon cancer reported, constituting 10.2% of all cancer cases. This malignancy, standing as the second most common cancer worldwide, follows closely behind lung and breast cancer. Furthermore, the associated mortality figures are equally concerning, with over 840,000 deaths attributed to colon cancer, accounting for 9.2% of all cancer-related deaths (1).

In the secondary analysis of global cancer statistics for 2020, colon cancer maintains its significant impact, ranking second in incidence and fifth in mortality within China (2). Despite advancements in systemic therapy that have contributed to a decline in the incidence of distant metastasis in colon cancer, postoperative local recurrence rates remain notable, ranging between 10% and 40% (3). This underscores the imperative for effective postoperative local treatments, such as radiotherapy (4, 5). Traditionally, adjuvant radiotherapy is not routinely recommended for COAD and is typically reserved for specific clinical scenarios, including locally advanced disease (pT4) and/or positive margins (6). However, due to the limited utilization of radiotherapy in clinical practice and the scarcity of comprehensive clinical trials, the therapeutic efficacy of radiotherapy in COAD remains uncertain (7).

Recent updates in the 2020 NCCN guidelines mark a subtle expansion in the indications for adjuvant radiotherapy. Remarkably, individuals with a confirmed postoperative T4 stage with fixation are currently being contemplated for radiotherapy, albeit with a class II recommendation. Nevertheless, the influence of adjuvant radiotherapy on the overall prognosis of patients with COAD remains elusive.

In light of this, our retrospective study utilized the Surveillance, Epidemiology, and End Results (SEER) database to analyze the survival outcomes of patients with pT4M0 COAD who underwent various treatment modalities. The study aimed to elucidate prognostic factors influencing the outcomes of these patients and, subsequently, construct and validate nomograms to predict overall survival (OS) and cancer-specific survival (CSS).

## 2 Materials and methods

### 2.1 Study population

The inclusion criteria for patients in the SEER database search were as follows (1): patients diagnosed with COAD as their initial malignancy between January 2010 and December 2015 (2); T4 and M0 staging according to the American Joint Committee on Cancer 7th Edition Staging System (3); definitive cause of death and treatment details, including initial surgery or external radiotherapy, with or without chemotherapy; and (4) survival time of at least 1 month. Ultimately, this study included 8,843 patients with stage pT4M0 COAD. All treatments, including surgery, radiotherapy, and chemotherapy, were administered as the initial treatment upon diagnosis.

### 2.2 Treatment groups

All patients underwent surgery. Patients exclusively undergoing surgery were included in the S group. Patients receiving postoperative adjuvant radiotherapy were included in the S+R group. Those receiving postoperative adjuvant chemotherapy were included in the S+C group. Patients undergoing postoperative adjuvant chemoradiotherapy were included in the S+R+C group. For subsequent analysis convenience, the S+R group, S+C group, and S+R+C group were combined into the surgery with the postoperative adjuvant therapy (S+ADT) group.

### 2.3 Statistical analysis

The study delineated OS as the duration from randomization to death from any cause. CSS was specifically defined as the duration from randomization to death caused by COAD. OS was designated as the primary endpoint, with CSS serving as the secondary endpoint.

The statistical analysis was performed using SPSS 26.0 and R-Software 4.1.2, and graphs were generated through R packages, including ‘survival,’ ‘timeROC,’ ‘ggDCA,’ ‘dplyr,’ and ‘rms.’ Pearson’s chi-square test was employed to compare the characteristics of different treatment groups. Univariate and multivariate analyses utilized COX proportional hazards models to assess and compare the prognostic significance of clinicopathologic variables on OS and CSS rates.

The Kaplan-Meier method, followed by a log-rank test, was employed to analyze survival curves. Variables found significant in the univariate analysis were included in the multivariate analysis to construct the nomogram. Statistical significance was set at P < 0.05.

The accuracy of the nomogram was evaluated using the C-index, receiver operating characteristic (ROC) curve, and calibration curve. Clinical usefulness and benefits were estimated using decision curve analysis (DCA) plots. Additionally, using risk score and X-tile software version 3.6.1 (Yale University, New Haven, CT), patients were stratified into low-, intermediate-, and high-risk groups.

## 3 Results

### 3.1 Patient characteristics

We identified 10,041 patients diagnosed with pT4M0 colon cancer between January 2010 and December 2015 from the SEER database. Exclusions were applied to 649 patients with a pathological type other than adenocarcinoma, 491 based on treatment modality, and 59 with a survival period of less than 1 month. This resulted in a final cohort of 8,843 patients for the current analysis. The entire SEER cohort was randomly divided into training and validation sets in a 7:3 ratio, and summarized characteristics are provided in **Table 1**.

**Table 1:** Clinical data of the study subjects.

	Overall	Training set	Validation set	P
	N=8843 NO.(%)	N=6190 NO.(%)	N=2653 NO.(%)	
Age (%)				
<50	1049 (11.86)	729 (11.78)	320 (12.06)	0.577
50~75	3119 (35.27)	2166 (34.99)	953 (35.92)	
≥75	4675 (52.87)	3295 (53.23)	1380 (52.02)	
Sex (%)				
Female	4500 (50.89)	3156 (50.99)	1344 (50.66)	0.797
Male	4343 (49.11)	3034 (49.01)	1309 (49.34)	
Race (%)				
White	6937 (78.45)	4878 (78.80)	2059 (77.61)	0.619
Black	909 (10.28)	622 (10.05)	287 (10.82)	
Other	973 (11.00)	674 (10.89)	299 (11.27)	
Unknown	24 (0.27)	16 (0.26)	8 (0.30)	
Primary site (%)				
Ascending Colon	2130 (24.09)	1495 (24.15)	635 (23.94)	0.987
Hepatic Flexure	547 (6.19)	377 (6.09)	170 (6.41)	
Transverse Colon	1294 (14.63)	901 (14.56)	393 (14.81)	
Splenic Flexure	492 (5.56)	347 (5.61)	145 (5.47)	
Descending Colon	790 (8.93)	546 (8.82)	244 (9.20)	
Descending Colon	3191 (36.09)	2245 (36.27)	946 (35.66)	
Large Intestine	399 (4.51)	279 (4.51)	120 (4.52)	
Grade (%)				
I	417 (4.72)	321 (5.19)	96 (3.62)	0.021
II	5621 (63.56)	3936 (63.59)	1685 (63.51)	
III	2083 (23.56)	1430 (23.10)	653 (24.61)	
IV	458 (5.18)	318 (5.14)	140 (5.28)	
unknow	264 (2.99)	185 (2.99)	79 (2.98)	
NStage (%)				
N0	3766 (42.59)	2628 (42.46)	1138 (42.89)	0.855
N1	2978 (33.68)	2102 (33.96)	876 (33.02)	
N2	2042 (23.09)	1420 (22.94)	622 (23.45)	
NX	57 (0.64)	40 (0.65)	17 (0.64)	
TStage (%)				
T4	38 (0.43)	22 (0.36)	16 (0.60)	0.259
T4a	5526 (62.49)	3867 (62.47)	1659 (62.53)	
T4b	3279 (37.08)	2301 (37.17)	978 (36.86)	
Treatment (%)				
S	4215 (47.66)	2980 (48.14)	1235 (46.55)	0.575
S+C	4248 (48.04)	2945 (47.58)	1303 (49.11)	
S+R	49 (0.55)	35 (0.57)	14 (0.53)	
S+R+C	331 (3.74)	230 (3.72)	101 (3.81)	
CEA (%)				
	2579 (29.16)	1808 (29.21)	771 (29.06)	0.974
abnormal	2838 (32.09)	1982 (32.02)	856 (32.27)	
unknown	3426 (38.74)	2400 (38.77)	1026 (38.67)	
Node removed (%)				
normal	1435 (16.23)	1017 (16.43)	418 (15.76)	0.515
abnormal	7363 (83.26)	5139 (83.02)	2224 (83.83)	
unknown	45 (0.51)	34 (0.55)	11 (0.41)	
Size (%)				
<5	3355 (37.94)	2347 (37.92)	1008 (37.99)	0.679
≥5	5154 (58.28)	3602 (58.19)	1552 (58.50)	
unknown	334 (3.78)	241 (3.89)	93 (3.51)	

S, Surgery; S+R, Surgery+Radiotherapy; S+C, Surgery+Chemotherapy; S+R+C, Surgery+Radiotherapy+Chemotherapy.

**Figure 1:**
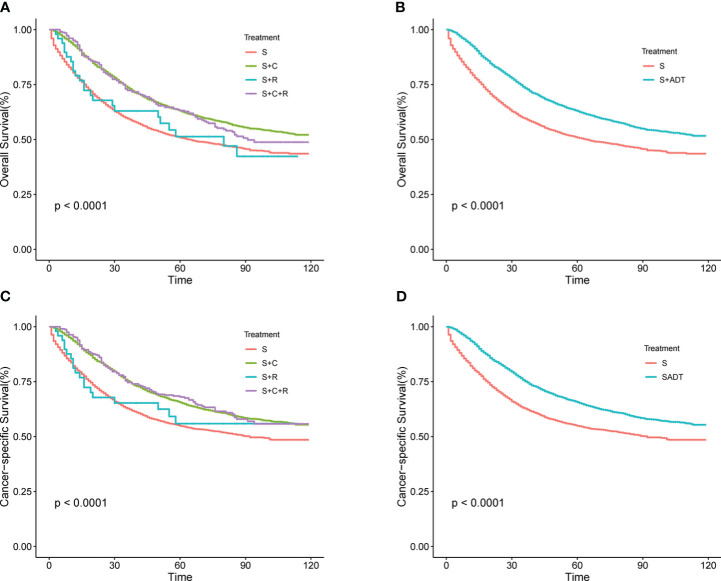
**(A–D)** Survival rates for T4M0 COAD patients grouped by treatment throughout the cohort. **(A)** OS rates between four groups **(B)** OS rates between S and S+ ADT groups **(C)** CSS rates between four groups **(D)** CSS rates between S and S+ADT groups.

In the total SEER cohort, the median survival for the overall population was 49 (1–119) months, with 1-, 3-, and 5-year OS rates of 86.63%, 68.97%, and 61.19%, respectively. Corresponding CSS rates were 87.89%, 71.83%, and 65.01%. Within the S+C and S+R+C groups, 1-, 3-, and 5-year OS rates were 92.47% vs. 94.56%, 74.41% vs. 73.11%, and 65.16% vs. 64.95%, respectively. For CSS, the rates were 93.08% vs. 95.17%, 76.39% vs. 75.83%, and 61.89% vs. 70.09%. Survival curves for the S+C and S+R+C groups (**Figures 1A, C**) were similar and superior to those in the S group. Due to limited cases in the S+R and S+R+C groups, they were combined into the S+ADT group. The 1-year OS rate for the S+ADT group was 92.48%, compared to 80.21% in the S group. At 3 years, the rates were 74.22% vs. 63.20%, and at 5 years, 65.06% vs. 56.94%, all significantly better in the S+ADT group (P<0.01). The 1-, 3-, and 5-year CSS rates (93.09%, 76.25%, and 68.13%) in the S+ADT group surpassed those in the S group (**Figure 1**). These findings strongly supported the recommendation of surgery with postoperative adjuvant therapy for pT4M0 COAD.

### 3.2 Independent prognostic predictors of OS and CSS

Univariate COX regression analysis during training revealed significant influences on OS, including age, race, primary site, degree of differentiation, N stage, serum carcinoembryonic antigen (CEA), tumor size, and number of lymph nodes resected. Similarly, age, race, primary site, gender, degree of differentiation, N stage, serum CEA, tumor size, and the number of lymph nodes resected were associated with CSS (**Table 2**). In a multifactorial COX regression analysis adjusting for covariates, independent predictors of both OS and CSS included age, race, differentiation grade, N stage, serum CEA, tumor size, and the number of resected lymph nodes (**Table 3**).

**Table 2:** Univariate COX regression analysis of OS and CSS.

	Univariate COX regression analysis					
Variable	OS			CSS		
	HR	95%CI	P	HR	95%CI	P
Age						
<50						
50~75	1.279	1.114-1.468	0.000	1.253	1.084-1.448	0.002
≥75	2.199	1.911-2.531	0.000	2.176	1.879-2.521	0.000
Race						
White						
Black	1.134	0.945-1.211	0.288	0.798	0.670-0.951	0.012
Other	0.786	0.735-0.951	0.006	0.255	0.064-1.026	0.054
Unknown	0.243	0.062-0.992	0.049	0.921	0.808-1.049	0.215
Sex						
Female						
Male	0.926	0.857-1.000	0.050	0.921	0.849-0.999	0.047
Primary site						
Ascending Colon						
Hepatic Flexure	1.118	0.942-1.325	0.202	1.139	0.952-1.361	0.154
Transverse Colon	0.965	0.847-1.100	0.595	0.970	0.846-1.112	0.662
Splenic Flexure	0.985	0.823-1.180	0.872	0.974	0.805-1.179	0.789
Descending Colon	0.965	0.829-1.122	0.641	0.978	0.835-1.146	0.786
Sigmoid Colon	0.937	0.847-1.037	0.21	0.924	0.83-1.029	0.150
Large Intestine	1.258	1.041-1.519	0.017	1.301	1.069-1.582	0.009
Grade						
I						
II	0.985	0.821-1.181	0.871	1.031	0.849-1.253	0.757
III	1.440	1.191-1.741	0.000	1.49	1.215-1.826	0.000
IV	1.650	1.308-2.083	0.000	1.719	1.342-2.203	0.000
unknown	2.022	1.559-2.621	0.000	2.065	1.564-2.726	0.000
N Stage						
N0						
N1	1.355	1.235-1.488	0.000	1.380	1.250-1.523	0.000
N2	2.059	1.871-2.267	0.000	2.082	1.881-2.305	0.000
NX	5.578	3.938-7.901	0.000	5.677	3.940-8.181	0.000
Treatment						
S						
S+C	0.683	0.631-0.74	0.000	0.709	0.652-0.770	0.000
S+R	1.085	0.672-1.75	0.739	1.081	0.650-1.799	0.764
S+R+C	0.745	0.608-0.913	0.005	0.733	0.589-0.911	0.005
CEA						
normal						
abnormal	1.591	1.438-1.761	0.000	1.587	1.426-1.767	0.000
unknown	1.431	1.296-1.580	0.000	1.440	1.297-1.599	0.001
Node removed						
<12						
≥12	0.518	0.472-0.568	0.000	0.517	0.468-0.570	0.000
unknown	0.982	0.635-1.518	0.934	0.984	0.622-1.555	0.984
Size						
<5						
≥5	1.068	0.986-1.156	0.012	1.094	1.004-1.191	0.041
unknown	1.914	1.611-2.274	0.000	2.181	1.825-2.606	0.000

**Table 3:** Multivariate COX regression analysis of OS and CSS.

	Multivariate COX regression analysis					
Variable	OS			CSS		
	HR	95%CI	P	HR	95%CI	P
Age						
<50						
50~75	1.189	1.035-1.368	0.015	1.172	1.012-1.356	0.034
≥75	1.932	1.665-2.241	0.000	1.943	1.662-2.272	0.000
Race						
White						
Black	1.108	0.976-1.258	0.114	1.125	0.984-1.286	0.084
Other	0.775	0.681-0.883	0.000	0.805	0.703-0.921	0.002
Unknown	0.216	0.054-0.866	0.031	0.245	0.061-0.985	0.047
Grade						
I						
II	1.003	0.836-1.204	0.973	1.046	0.86-1.272	0.655
III	1.266	1.044-1.535	0.017	1.299	1.056-1.597	0.013
IV	1.507	1.191-1.906	0.001	1.555	1.21-1.997	0.001
unknown	1.361	1.036-1.789	0.027	1.374	1.026-1.839	0.033
N Stage						
N0						
N1	1.600	1.452-1.763	0.000	1.611	1.455-1.875	0.000
N2	2.671	2.410-2.960	0.000	2.670	2.394-2.977	0.000
NX	2.311	1.582-3.377	0.000	2.291	1.539-3.41	0.000
Treatment						
S						
S+C	0.666	0.61-0.727	0.000	0.696	0.635-0.764	0.000
S+R	1.246	0.770-2.015	0.370	1.269	0.760-2.117	0.362
S+R+C	0.842	0.684-1.038	0.107	0.844	0.675-1.055	0.136
CEA						
normal						
abnormal	1.526	1.378-1.690	0.000	1.520	1.364-1.693	0.000
unknown	1.310	1.185-1.448	0.000	1.321	1.189-1.469	0.000
node removed						
<12						
≥12	0.505	0.458-0.558	0.000	0.505	0.455-0.56	0.000
unknown	0.720	0.461-1.124	0.148	0.703	0.44-1.123	0.140
Size						
<5						
≥5	1.158	1.065-1.258	0.001	1.145	1.048-1.25	0.003
unknown	1.839	1.515-2.233	0.000	1.907	1.559-2.333	0.000

### 3.3 Construction and validation of the nomogram

**Figure 2:**
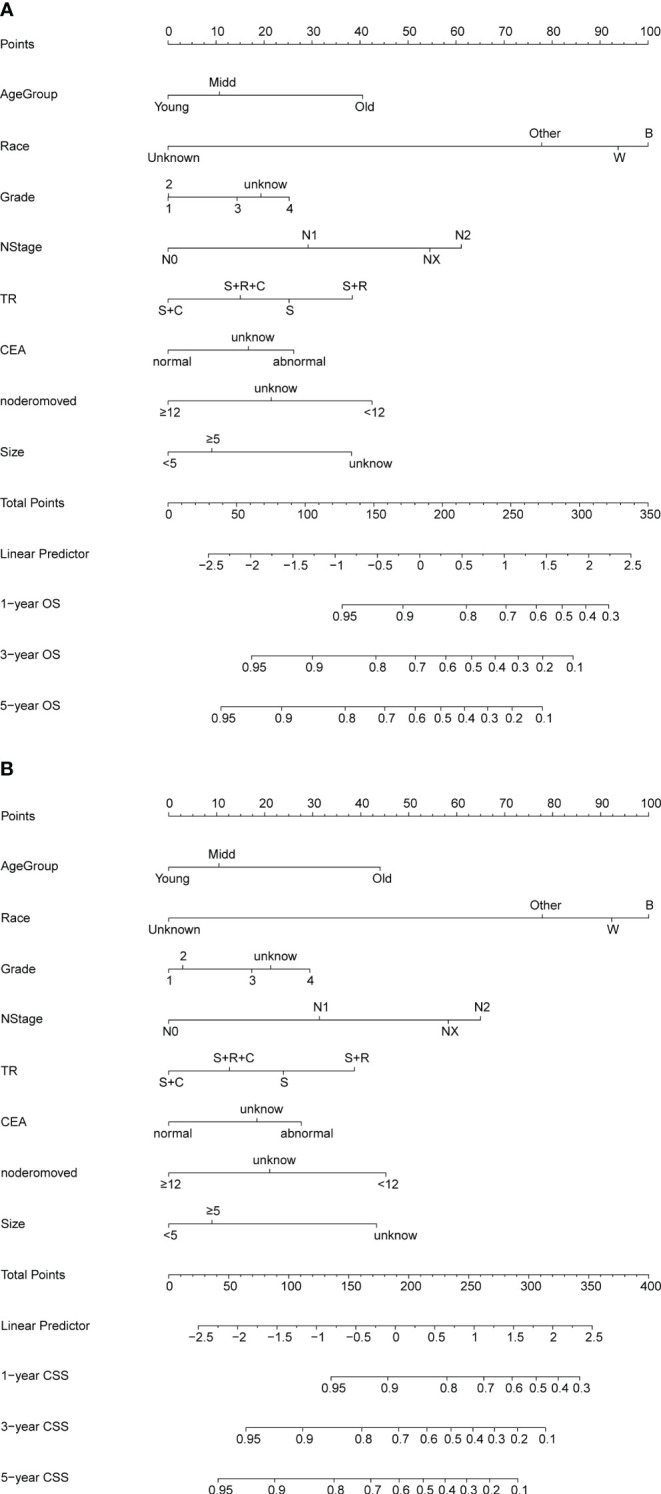
**(A, B)** Nomogram for predicting OS and CSS of patients with PT4M0 COAD. **(A)** Nomogram for predicting 1-, 3-, and 5-year OS; **(B)** Nomogram for predicting 1-, 3-, and 5-year CSS.

Nomograms for OS and CSS were developed using independent predictors identified through multifactorial COX regression analysis in the training set (**Figure 2**). The nomograms highlighted race as the most significant factor impacting OS, followed by N stage, treatment modality, number of resected lymph nodes, age, tumor size, serum CEA levels, and degree of differentiation. Similarly, for CSS, race emerged as the foremost influential factor, succeeded by N stage, number of resected lymph nodes, age, tumor size, treatment modality, degree of differentiation, and serum CEA levels. The R2 values for the OS and CSS models were 0.148 and 0.134, respectively (**Supplementary Table 4**).

**Figure 3:**
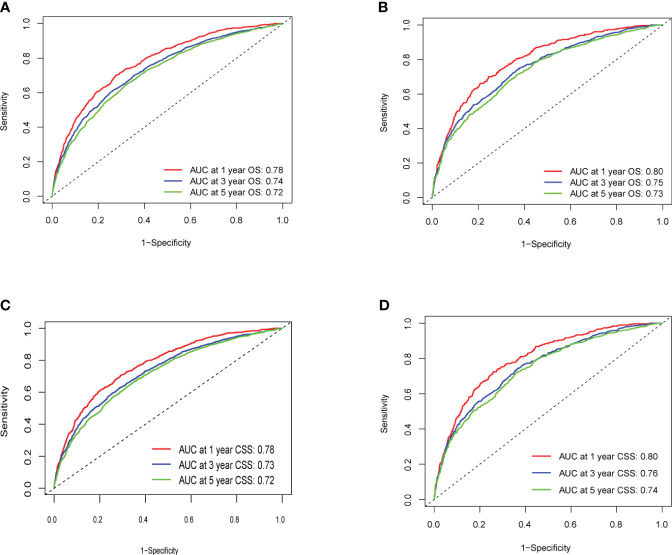
**(A–D)** ROC Curve of OS and CSS of Training Group and Validation Group. **(A)** Training Group OS; **(B)** Validation Group OS; **(C)** Training Group CSS; **(D)** Validation Group CSS.

The C-index values for predicting OS and CSS in the training set were 0.692 and 0.690, respectively. In the internal validation set, these values improved to 0.703 and 0.708, respectively (**Figure 3**), indicating commendable accuracy. The area under the curve (AUC) for 1-, 3-, and 5-year OS in the training set was 0.78, 0.74, and 0.72, respectively. In the validation set, these figures were 0.80, 0.75, and 0.73, respectively. As for CSS, the AUC for 1-, 3-, and 5-year OS in the training set was 0.78, 0.73, and 0.72, respectively. In the validation set, these AUCs were 0.80, 0.76, and 0.74, respectively.

**Figure 4:**
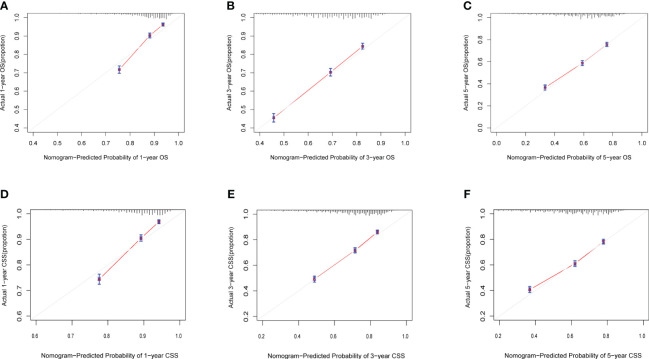
**(A–F)** Calibration curves of training group 1-, 3-, and 5-year OS and CSS. **(A)** Training group 1-year OS; **(B)** Training group 3-year OS; **(C)** Training group 5-year OS; **(D)** Training group 1-year CSS; **(E)** Training group 3-year CSS; **(F)** Training group 5-year CSS.

**Figure 5:**
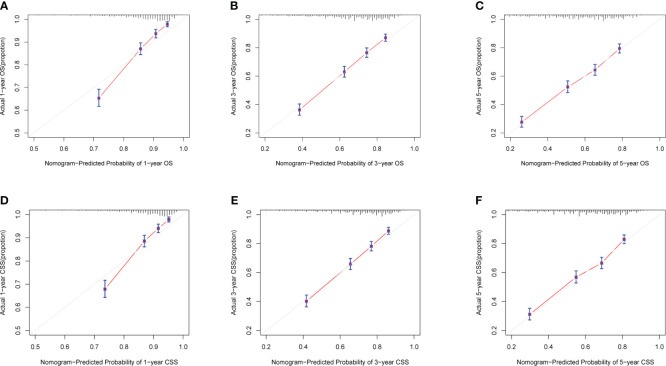
**(A-F)** Calibration curves of validation group 1-, 3-, and 5-year OS and CSS. **(A)** Validation group 1-year OS; **(B)** Training group 3-year OS; **(C)** Validation group 5-year OS; **(D)** Validation group 1-year CSS; **(E)** Validation group 3-year CSS; **(F)** Validation group 5-year CSS.

**Figure 6:**
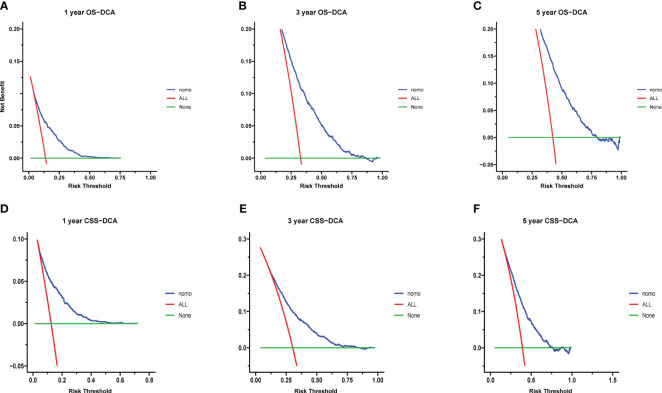
**(A–F)** Decision curves of training group 1-, 3-, and 5-year OS and CSS. **(A)** 1-year OS; **(B)** 3-year OS; **(C)** 5-year OS; **(D)** 1-year CSS; **(E)** 3-year CSS; **(F)** 5-year CSS.

**Figure 7:**
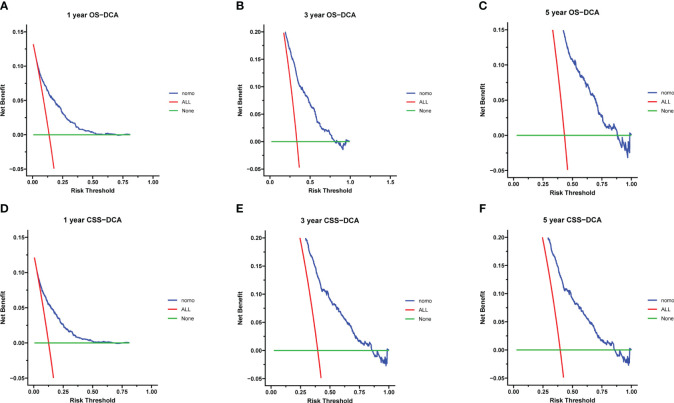
**(A–F)** Decision curves of validation group 1-, 3-, and 5-year OS and CSS **(A)** 1-year OS; **(B)** 3-year OS; **(C)** 5-year OS; **(D)** 1-year CSS; **(E)** 3-year CSS; **(F)** 5-year CSS.

Calibration curves for predicting 1-, 3-, and 5-year OS and CSS exhibited no deviation from the 45-degree diagonal lines in both the training set and the validation set (**Figures 4**, **5**), signifying a high level of agreement between predicted and observed outcomes. Clinical DCA confirmed the robust clinical applicability of the nomograms in predicting 1-, 3-, and 5-year OS and CSS in both the training set (**Figure 6**) and the validation set (**Figure 7**).

**Figure 8:**
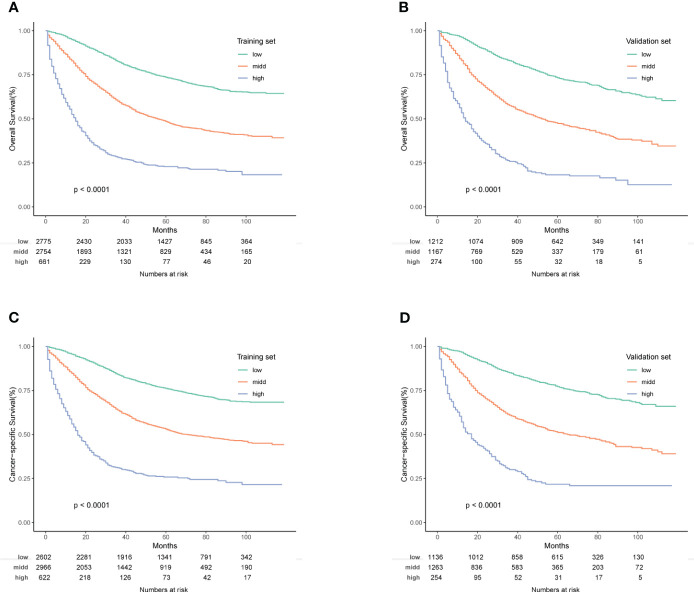
**(A–D)** Kaplan-Meier OS and CSS survival curves for different risk groups in the training and validation groups. **(A)** Training group of OS; **(B)** Validation group of OS; **(C)** Training group of CSS; **(D)** Validation group of CSS.

In conclusion, risk scores, computed through the nomogram, facilitated effective risk stratification. Patients were stratified into three risk subgroups based on cutoff values determined by X-tile software. For the OS nomogram, patients fell into low risk (points ≤ 176.28), intermediate risk (176.28 < points ≤ 236.60), and high risk (points > 236.60) categories. Similarly, the CSS nomogram classified patients into three risk categories: low risk (points ≤ 178.67), intermediate risk (178.67 < points ≤ 245.69), and high risk (points > 245.69). Kaplan-Meier survival curves depicted distinct differentiation among the various risk subgroups (**Figure 8**).

### 3.4 Subgroup analysis

**Figure 9:**
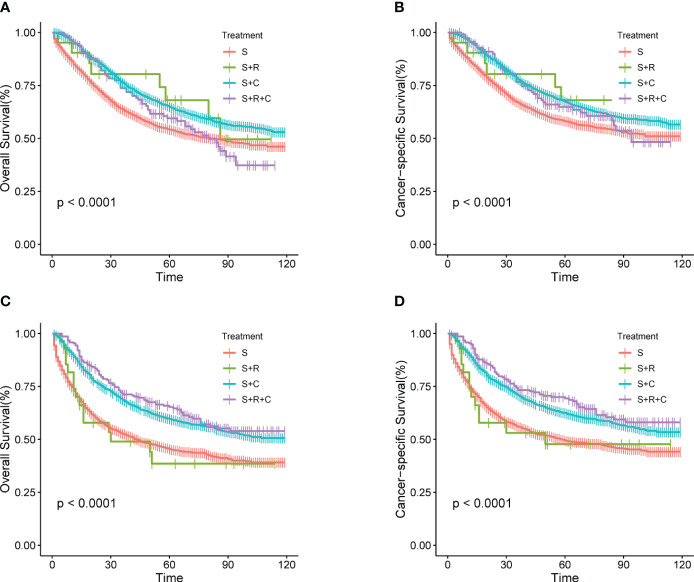
**(A–D)** Kaplan-Meier Survival curves of OS and CSS for stage pT4aM0 and pT4bM0 COAD comparing different treatments. **(A)** pT4aM0 group of OS; **(B)** pT4aM0 group of CSS; **(C)** pT4bM0 group of OS; **(D)** pT4bM0 group of CSS.

As depicted in **Figure 9**, Kaplan-Meier analysis of the treatment group within the pT4aM0 subgroup revealed a more favorable prognosis for the S+R group. Conversely, in the T4bM0 subgroup, Kaplan-Meier analysis demonstrated that the S+R group exhibited similar OS and CSS rates compared to the S group. This suggested that postoperative radiotherapy might confer greater benefits to patients with pT4aM0 COAD. Single and multiple COX regression analyses for OS and CSS were performed for various variables in the pT4aM0 and pT4bM0 subgroups, with results presented in **Supplementary Tables 2A, B** and **Supplementary Tables 3A, B**, respectively. Forest plots were generated to visualize the findings (**Supplementary Figures 1, 2**). The outcomes indicated that the S+C group derived benefits across all subgroups. However, a significant benefit was observed in the S+R+C group within the pT4bM0 subgroup, while no such benefit was evident in the pT4aM0 subgroup.

## 4 Discussion

The primary recommended treatment for pT4M0 COAD currently involves surgery followed by adjuvant chemotherapy, with adjuvant radiotherapy not considered a standard approach. Early studies (8, 9) indicating improved local control and disease-free survival (DFS) through radiotherapy in locally advanced COAD date back to the 1980s and 1990s. While many studies suggest enhanced survival with postoperative radiation therapy, the absence of supportive phase III trials has limited its acceptance for COAD. Notably, the only randomized controlled phase III trial has failed to establish a role for adjuvant radiotherapy (10).

The prognostic impact of radiotherapy on COAD remains unclear, and its application is generally discouraged in clinical practice. Owing to the limited availability of clinical data, prior studies in this domain often resort to analyzing information from large public databases such as the National Cancer Database (NCDB) and the SEER databases (11–14).

In this study, we conducted a Kaplan-Meier survival a0alysis for the S group, S+C group, S+R group, and S+R+C group, revealing a significant difference (P<0.05). Notably, the S+R+C group exhibited the highest OS rate and CSS rate. The S+C group demonstrated the second-highest rates, while the S group had the lowest rates. However, survival analysis between the S+C and S+R+C groups did not reveal statistically significant differences. A phase III trial has indicated similar OS and DFS for patients receiving chemotherapy, with higher toxicity observed in those undergoing single chemoradiotherapy (10).

Several studies, have investigated the progression of COAD, suggesting that the critical period for progression often occurs within 3 years post-surgery (15, 16). Our study aligned with these findings, revealing a 3-year OS of 68.97% and a 1-year OS of 86.63% for patients with pT4M0 COAD. The 3-year CSS was 71.83%, and the 1-year CSS was 87.89%.

The determinants of prognosis for COAD remain inconclusive, with varied results across studies. Wang et al. (17) have emphasized the significance of the tumor primary site, T stage, and serum CEA level, while Vergara-Fernandez et al. (18) have underscored the importance of the number of resected lymph nodes and nerve invasion. This complexity suggests that the recurrence of COAD metastasis is likely influenced by multiple and intricate factors.

To predict OS and CSS, we constructed nomograms based on multifactorial COX regression analysis, incorporating factors such as age, race, degree of differentiation, N stage, serum CEA levels, tumor size, and the number of resected lymph nodes. Validation was performed using calibration curves, ROC curves, and DCA, with further confirmation from an independent validation group.

Our findings revealed that age independently impacted prognosis, with 52.87% of patients aged ≥75 years in the entire SEER cohort. Patients in this age group faced a more than twofold higher risk of death compared to those aged <50 years (HR = 1.943, 95% CI: 1.662-2.272, P < 0.001), likely associated with poorer health status and a higher prevalence of comorbidities, consistent with previous studies (5, 6, 11, 19).

Serum CEA level was routinely used as an indicator for diagnosing and monitoring COAD (20–22). In our study, elevated serum CEA emerged as an independent risk factor for prognosis (HR = 1.319, 95% CI: 1.186-1.466, P = 0.000). Although serum CEA levels can rise in various malignant tumors and inflammatory or degenerative diseases, our study supported its role as an independent prognostic factor.

N stage, reflecting the extent of local advancement, was a significant prognostic factor. Patients with stage N1 faced a 1.611 times higher risk of death than those with stage N0 (95% CI: 1.455-1.875, P = 0.000), while stage N2 patients had a 2.67 times higher risk (95% CI: 2.394-2.977, P = 0.000). The number of resected lymph nodes, with a threshold of 12, influenced prognosis, with better outcomes for patients with ≥12 lymph nodes resected compared to those with <12 (P < 0.001, 95% CI: 0.455-0.560), consistent with prior studies (4, 18, 23, 24).

Tumor size ≥5 cm was associated with a 1.145 times higher risk of death than sizes <5 cm (95% CI: 1.048-1.25, P = 0.003). Pathopathological grades III and IV carried a higher risk of death compared to grade I (HRs: 1.299 vs. 1.555, P < 0.05), while the risk in grade II, though higher than grade I, did not reach statistical significance (P = 0.655).

In contrast to previous analyses of COAD prognosis, this study focused on a survival analysis of the treatment modality. While the radiotherapy group had a relatively small number of cases in the survival analysis, there was only a slight difference in the distribution of baseline clinical characteristics of the data (P>0.05), and therefore, propensity score matching (PSM) was not performed.

Acknowledging certain limitations in our study is essential. Being a retrospective study, it is susceptible to selection bias between groups. First, the information in the SEER database, collected by a single center, did not provide insight into whether patients received subsequent treatment at other facilities, potentially impacting their survival time. Second, the database lacked detailed information on factors such as physical status, CEA expression level, radiotherapy dose, chemotherapy regimen, and infiltration depth, which could enhance the accuracy of diagnostic and prognostic models. Third, the SEER database did not furnish comprehensive details about patients’ underlying diseases, such as severe coronary heart disease, liver and kidney diseases, or diabetes, which play a pivotal role in treatment decisions. Lastly, the patients included in the SEER database are predominantly from the United States, raising the question of the generalizability of the results to the Chinese population. Our study lacked Chinese patients for external validation. Notably, according to the modeling in this study, race independently influenced OS and CSS. Consequently, large-scale randomized controlled trials (RCTs) conducted in China are imperative to validate the potential benefits of postoperative adjuvant chemoradiotherapy.

## 5 Conclusion

For patients with COAD at the pT4M0 stage, the combination of surgery and adjuvant chemoradiotherapy demonstrated a significant extension of long-term survival. The nomogram, incorporating variables such as age, race, degree of differentiation, N stage, serum CEA level, tumor size, and the number of resected lymph nodes, stood as a reliable tool for predicting OS and CSS rates in this specific cohort. The utilization of this nomogram can prove instrumental for clinicians in identifying high-risk patients and formulating personalized treatment plans tailored to the unique characteristics of individuals with pT4M0 COAD.

## Data availability statement

The original contributions presented in the study are included in the article/**Supplementary Material**. Further inquiries can be directed to the corresponding author.

## Author contributions

XZ: Data curation, Software, Writing – original draft. LH: Methodology, Supervision, Writing – review & editing. QM: Data curation, Writing – review & editing. MZ: Data curation, Writing – review & editing. JL: Writing – review & editing.
