# Supplementary material for: Optimal treatment strategy and prognostic analysis for patients with non-metastatic pT4 colon adenocarcinoma
Source: Front Oncol. 2024 Jan 8;13:1342289. doi: 10.3389/fonc.2023.1342289 (PMC10802841; doi:10.3389/fonc.2023.1342289)
Supplement: Supplementary file 1 [file DataSheet_1.docx]

**Supplementary Table S1** The beta coefficients of the OS and CSS models

**Supplementary Table S2a** Univariate and multivariable COX regression analysis of T4a OS

**Supplementary Table S2b** Univariate and multivariable COX regression analysis of T4a CSS

**Supplementary Table S3a** Univariate and multivariable COX regression analysis of T4b OS

**Supplementary Table S3b** Univariate and multivariable COX regression analysis of T4b CSS

**Supplementary Figure S1** Forest plots of prognosis analysis of OS and CSS of pT4aM0 COAD. (A) OS of pT4aM0 COAD; (B) CSS of pT4aM0 COAD

**Supplementary Figure S2** Forest plots of prognosis analysis of OS and CSS of pT4bM0 COAD. (A) OS of pT4bM0 COAD; (B) CSS of pT4bM0 COAD

HR, hazard ratio; CI, confidential interval

**Table S1 The beta coefficients of the OS and CSS models**

|  | OS model | CSS model |
| --- | --- | --- |
| Age Group=Midd | 0.1740 | 0.1594 |
| Age Group=Old | 0.6581 | 0.6704 |
| Race=Black | 0.1010 | 0.1175 |
| Race=Other | -0.2588 | -0.2202 |
| Race=Unknown | -1.5219 | -0.4053 |
| Grade=2 | 0.0014 | 0.0443 |
| Grade=3 | 0.2332 | 0.2634 |
| Grade=4 | 0.4097 | 0.4477 |
| Grade=unknow | 0.3142 | 0.3237 |
| N Stage=N1 | 0.4732 | 0.4781 |
| N Stage=N2 | 0.9917 | 0.9891 |
| N Stage=NX | 0.8851 | 0.8865 |
| TR=S+R | 0.2137 | 0.2255 |
| TR=S+C | -0.4093 | -0.3634 |
| TR=S+R+C | -0.3410 | -0.1708 |
| CEA=Abnormal | 0.1479 | 0.4205 |
| CEA=Abnormal | 0.2711 | 0.2799 |
| Node romoved=≥12 | –0.6899 | -0.6888 |
| Node romoved=unknow | -0.3410 | -0.3684 |
| Size=≥5 | 0.1479 | 0.1370 |
| Size=unknow | 0.6205 | 0.6595 |

**Table S2a Univariate and Multivariable COX regression analysis of T4a OS**

| Variable | Univariate analysis | | | Multivariable analysis | | |
| --- | --- | --- | --- | --- | --- | --- |
| T4aOS | HR | 95%CI | P | HR | 95%CI | P |
| Age |  |  |  |  |  |  |
| <50 |  |  |  |  |  |  |
| 50~75 | 1.200 | 1.035-1.390 | 0.016 | 1.179 | 1.015-1.369 | 0.031 |
| ≥75 | 2.114 | 1.820-2.456 | 0.000 | 1.931 | 1.645-2.266 | 0.000 |
| Race |  |  |  |  |  |  |
| White |  |  |  |  |  |  |
| Black | 1.074 | 0.939-1.230 | 0.298 | 1.118 | 0.974-1.282 | 0.112 |
| Other | 0.834 | 0.730-0.952 | 0.007 | 0.771 | 0.674-0.882 | 0.000 |
| Unknown | 0.159 | 0.022-1.127 | 0.066 | 0.152 | 0.021-1.084 | 0.060 |
| Sex |  |  |  |  |  |  |
| Female |  |  |  |  |  |  |
| Male | 0.901 | 0.901-0.979 | 0.014 | 0.953 | 0.875-1.037 | 0.262 |
| Primary site |  |  |  |  |  |  |
| Ascending Colon |  |  |  |  |  |  |
| Hepatic Flexure | 1.214 | 1.008-1.462 | 0.040 | 1.139 | 1.035-1.502 | 0.020 |
| Transverse Colon | 1.102 | 0.960-1.265 | 0.167 | 0.970 | 0.976-1.288 | 0.106 |
| Splenic Flexure | 0.946 | 0.770-1.162 | 0.595 | 0.974 | 0.807-1.222 | 0.948 |
| Descending Colon | 0.966 | 0.824-1.132 | 0.668 | 0.978 | 0.921-1.273 | 0.335 |
| Sigmoid Colon | 0.994 | 0.892-1.108 | 0.920 | 0.924 | 0.996-1.246 | 0.060 |
| Large Intestine | 1.306 | 1.052-1.621 | 0.016 | 1.301 | 0.946-1.461 | 0.145 |
| Grade |  |  |  |  |  |  |
| Ⅰ |  |  |  |  |  |  |
| Ⅱ | 0.985 | 0.804-1.201 | 0.867 | 0.998 | 0.816-1.22 | 0.981 |
| Ⅲ | 1.393 | 1.130-1.717 | 0.002 | 1.257 | 1.016-1.554 | 0.035 |
| Ⅳ | 1.674 | 1.302-2.151 | 0.000 | 1.486 | 1.154-1.914 | 0.002 |
| unknown | 1.137 | 0.772-1.676 | 0.516 | 1.086 | 0.733-1.609 | 0.681 |
| N Stage |  |  |  |  |  |  |
| N0 |  |  |  |  |  |  |
| N1 | 1.417 | 1.275-1.575 | 0.000 | 1.733 | 1.552-1.934 | 0.000 |
| N2 | 2.370 | 2.134-2.633 | 0.000 | 3.146 | 2.807-3.524 | 0.000 |
| NX | 3.558 | 1.144-11.064 | 0.028 | 1.902 | 0.598-6.052 | 0.276 |
| Treatment |  |  |  |  |  |  |
| S |  |  |  |  |  |  |
| S+R | 0.702 | 0.350-1.406 | 0.318 | 0.618 | 0.561-0.681 | 0.809 |
| S+C | 0.683 | 0.628-0.744 | 0.000 | 0.917 | 0.456-1.847 | 0.000 |
| S+R+C | 0.899 | 0.687-1.175 | 0.436 | 0.953 | 0.724-1.254 | 0.729 |
| CEA |  |  |  |  |  |  |
| normal |  |  |  |  |  |  |
| abnormal | 1.601 | 1.436-1.785 | 0.000 | 1.486 | 1.331-1.658 | 0.000 |
| unknown | 1.351 | 1.217-1.501 | 0.000 | 1.255 | 1.128-1.395 | 0.000 |
| Node removed |  |  |  |  |  |  |
| <12 |  |  |  |  |  |  |
| ≥12 | 0.687 | 0.614-0.769 | 0.000 | 0.645 | 0.574-0.724 | 0.000 |
| unknown | 1.831 | 1.030-3.254 | 0.039 | 1.436 | 0.802-2.569 | 0.223 |
| Size |  |  |  |  |  |  |
| <5 |  |  |  |  |  |  |
| ≥5 | 1.066 | 0.980-1.160 | 0.137 | 1.087 | 0.997-1.185 | 0.057 |
| unknown | 1.914 | 1.067-1.903 | 0.016 | 1.671 | 1.244-2.246 | 0.001 |

**Table S2b Univariate and Multivariable COX regression analysis of T4a CSS**

| Variable | Univariate analysis | | | Multivariable analysis | | |
| --- | --- | --- | --- | --- | --- | --- |
| T4aCSS | HR | 95%CI | P | HR | 95%CI | P |
| Age |  |  |  |  |  |  |
| <50 |  |  |  |  |  |  |
| 50~75 | 1.175 | 1.006-1.372 | 0.042 | 1.158 | 0.989-1.355 | 0.068 |
| ≥75 | 2.098 | 1.793-2.456 | 0.000 | 1.936 | 1.636-2.290 | 0.000 |
| Race |  |  |  |  |  |  |
| White | 1.072 | 0.929-1.237 | 0.339 |  |  |  |
| Black | 0.844 | 0.734-0.971 | 0.018 | 1.114 | 0.963-1.287 | 0.146 |
| Other | 0.177 | 0.025-1.259 | 0.084 | 0.781 | 0.678-0.900 | 0.001 |
| Unknown |  |  |  | 0.166 | 0.023-1.183 | 0.073 |
| Sex |  |  |  |  |  |  |
| Female |  |  |  |  |  |  |
| Male | 0.868 | 0.796-0.949 | 0.002 | 0.919 | 0.840-1.005 | 0.065 |
| Primary site |  |  |  |  |  |  |
| Ascending Colon |  |  |  |  |  |  |
| Hepatic Flexure | 1.249 | 1.027-1.519 | 0.026 | 1.282 | 1.054-1.559 | 0.013 |
| Transverse Colon | 1.143 | 0.988-1.322 | 0.072 | 1.159 | 1.001-1.341 | 0.048 |
| Splenic Flexure | 0.994 | 0.802-1232 | 0.958 | 1.051 | 0.846-1.304 | 0.655 |
| Descending Colon | 0.990 | 0.837-1.171 | 0.906 | 1.112 | 0.938-1.319 | 0.221 |
| Sigmoid Colon | 1.001 | 0.892-1.123 | 0.982 | 1.126 | 0.999-1.268 | 0.052 |
| Large Intestine | 1.313 | 1.043-1.653 | 0.020 | 1.189 | 0.943-1.498 | 0.142 |
| Grade |  |  |  |  |  |  |
| Ⅰ |  |  |  |  |  |  |
| Ⅱ | 1.027 | 0.828-1.273 | 0.811 | 1.035 | 0.834-1.285 | 0.752 |
| Ⅲ | 1.437 | 1.148-1.799 | 0.002 | 1.282 | 1.021-1.609 | 0.033 |
| Ⅳ | 1.6778 | 1.282-2.198 | 0.000 | 1.47 | 1.120-1.929 | 0.005 |
| unknown | 1.121 | 0.737-1.704 | 0.594 | 1.07 | 0.700-1.637 | 0.753 |
| N Stage |  |  |  |  |  |  |
| N0 |  |  |  |  |  |  |
| N1 | 1.460 | 1.305-1.633 | 0.000 | 1.768 | 1.574-1.987 | 0.000 |
| N2 | 2.412 | 2.158-2.698 | 0.000 | 3.183 | 2.821-3.592 | 0.000 |
| NX | 2.699 | 0.673-10.823 | 0.161 | 1.41 | 0.344-5.775 | 0.633 |
| Treatment |  |  |  |  |  |  |
| S |  |  |  |  |  |  |
| S+R | 0.597 | 0.267-1.331 | 0.207 | 0.808 | 0.361-1.811 | 0.605 |
| S+C | 0.705 | 0.644-0.771 | 0.000 | 0.64 | 0.577-0.708 | 0.000 |
| S+R+C | 0.799 | 0.590-1.080 | 0.144 | 0.858 | 0.630-1.167 | 0.329 |
| CEA |  |  |  |  |  |  |
| normal |  |  |  |  |  |  |
| abnormal | 1.604 | 1.430-1.798 | 0.000 | 1.481 | 1.319-1.663 | 0.000 |
| unknown | 1.34 | 1.200-1.498 | 0.000 | 1.245 | 1.112-1.392 | 0.000 |
| Node removed |  |  |  |  |  |  |
| <12 |  |  |  |  |  |  |
| ≥12 | 0.687 | 0.610-0.774 | 0.000 | 0.642 | 0.568-0.726 | 0.000 |
| unknown | 1.876 | 1.028-3.420 | 0.040 | 1.494 | 0.815-2.742 | 0.194 |
| Size |  |  |  |  |  |  |
| <5 |  |  |  |  |  |  |
| ≥5 | 1.060 | 0.969-1.159 | 0.201 | 1.089 | 0.994-1.193 | 0.067 |
| unknown | 1.461 | 1.080-1.977 | 0.014 | 1.755 | 1.289-2.389 | 0.000 |

**Table S3a Univariate and Multivariable COX regression analysis of T4b OS**

| Variable | Univariate analysis | | | Multivariable analysis | | |
| --- | --- | --- | --- | --- | --- | --- |
| T4bOS | HR | 95%CI | P | HR | 95%CI | P |
| Age |  |  |  |  |  |  |
| <50 |  |  |  |  |  |  |
| 50~75 | 1.337 | 1.115-1.603 | 0.002 | 1.219 | 1.014-1.466 | 0.035 |
| ≥75 | 2.502 | 2.081-3.008 | 0.000 | 1.989 | 1.639-2.414 | 0.000 |
| Race |  |  |  |  |  |  |
| White |  |  |  |  |  |  |
| Black | 1.161 | 0.995-1.354 | 0.058 | — | — | — |
| Other | 0.947 | 0.792-1.132 | 0.548 | — | — | — |
| Unknown | 0.149 | 0.021-1.058 | 0.057 | — | — | — |
| Sex |  |  |  |  |  |  |
| Female |  |  |  |  |  |  |
| Male | 0.976 | 0.882-1.079 | 0.633 | — | — | — |
| Primary site |  |  |  |  |  |  |
| Ascending Colon |  |  |  |  |  |  |
| Hepatic Flexure | 0.984 | 0.795-1.217 | 0.879 | 1.004 | 0.81-1.244 | 0.970 |
| Transverse Colon | 0.752 | 0.634-0.892 | 0.001 | 0.778 | 0.655-0.924 | 0.004 |
| Splenic Flexure | 0.884 | 0.708-1.105 | 0.280 | 0.99 | 0.792-1.239 | 0.932 |
| Descending Colon | 0.802 | 0.651-0.988 | 0.038 | 0.855 | 0.693-1.056 | 0.147 |
| Sigmoid Colon | 0.711 | 0.610-0.816 | 0.000 | 0.81 | 0.702-0.934 | 0.004 |
| Large Intestine | 0.973 | 0.770-1.229 | 0.819 | 0.845 | 0.667-1.072 | 0.166 |
| Grade |  |  |  |  |  |  |
| Ⅰ |  |  |  |  |  |  |
| Ⅱ | 1.046 | 0.805-1.360 | 0.734 | 1.048 | 0.806-1.363 | 0.726 |
| Ⅲ | 1.571 | 1.198-2.060 | 0.001 | 1.307 | 0.993-1.719 | 0.056 |
| Ⅳ | 1.873 | 1.354-2.589 | 0.000 | 1.727 | 1.243-2.400 | 0.001 |
| unknown | 2.429 | 1.772-3.330 | 0.000 | 1.527 | 1.101-2.117 | 0.011 |
| NStage |  |  |  |  |  |  |
| N0 |  |  |  |  |  |  |
| N1 | 1.434 | 1.275-1.614 | 0.000 | 1.586 | 1.405-1.79 | 0.000 |
| N2 | 1.810 | 1.588-2.063 | 0.000 | 2.190 | 1.908-2.515 | 0.000 |
| NX | 5.242 | 3.858-7.124 | 0.000 | 1.951 | 1.398-2.724 | 0.000 |
| Treatment |  |  |  |  |  |  |
| S |  |  |  |  |  |  |
| S+R | 1.051 | 0.631-1.752 | 0.848 | 1.266 | 0.758-2.114 | 0.368 |
| S+C | 0.615 | 0.554-0.683 | 0.000 | 0.656 | 0.585-0.735 | 0.000 |
| S+R+C | 0.545 | 0.435-0.681 | 0.000 | 0.64 | 0.508-0.807 | 0.000 |
| CEA |  |  |  |  |  |  |
| normal |  |  |  |  |  |  |
| abnormal | 1.526 | 1.335-1.745 | 0.000 | 1.437 | 1.255-1.646 | 0.000 |
| unknown | 1.404 | 1.227-1.608 | 0.000 | 1.255 | 1.094-1.439 | 0.001 |
| Node removed |  |  |  |  |  |  |
| <12 |  |  |  |  |  |  |
| ≥12 | 0.365 | 0.327-0.408 | 0.000 | 0.372 | 0.329-0.419 | 0.000 |
| unknown | 0.754 | 0.458-1.241 | 0.267 | 0.632 | 0.380-1.051 | 0.077 |
| Size |  |  |  |  |  |  |
| <5 |  |  |  |  |  |  |
| ≥5 | 0.893 | 0.785-1.016 | 0.086 | 0.966 | 0.847-1.102 | 0.609 |
| unknown | 1.947 | 1.604-2.363 | 0.000 | 1.397 | 1.121-1.740 | 0.003 |

**Table S3b Univariate and Multivariable COX regression analysis of T4b CSS**

| Variable | Univariate analysis | | | Multivariable analysis | | |
| --- | --- | --- | --- | --- | --- | --- |
|  | HR | 95%CI | P | HR | 95%CI | P |
| Age |  |  |  |  |  |  |
| <50 |  |  |  |  |  |  |
| 50~75 | 1.332 | 1.100-1.614 | 0.003 | 1.224 | 1.008-1.487 | 0.041 |
| ≥75 | 2.519 | 2.075-3.059 | 0.000 | 2.085 | 1.697-2.561 | 0.000 |
| Race |  |  |  |  |  |  |
| White |  |  |  |  |  |  |
| Black | 1.178 | 1.002-1.385 | 0.047 | 1.275 | 1.078-1.509 | 0.005 |
| Other | 0.994 | 0.827-1.196 | 0.951 | 0.972 | 0.805-1.172 | 0.763 |
| Unknown | 0.167 | 0.024-1.187 | 0.074 | 0.156 | 0.022-1.112 | 0.064 |
| Sex |  |  |  |  |  |  |
| Female |  |  |  |  |  |  |
| Male | 1.000 | 0.900-1.112 | 0.996 | — | — | — |
| Primary site |  |  |  |  |  |  |
| Ascending Colon |  |  |  |  |  |  |
| Hepatic Flexure | 0.98 | 0.786-1.224 | 0.861 | 1.011 | 0.809-1.264 | 0.920 |
| Transverse Colon | 0.740 | 0.619-0.885 | 0.001 | 0.761 | 0.636-0.912 | 0.003 |
| Splenic Flexure | 0.853 | 0.675-1.079 | 0.185 | 0.947 | 0.748-1.199 | 0.649 |
| Descending Colon | 0.824 | 0.665-1.021 | 0.077 | 0.866 | 0.697-1.077 | 0.195 |
| Sigmoid Colon | 0.670 | 0.580-0.774 | 0.000 | 0.764 | 0.657-0.888 | 0.000 |
| Large Intestine | 0.946 | 0.740-1.209 | 0.658 | 0.818 | 0.637-1.050 | 0.115 |
| Grade |  |  |  |  |  |  |
| Ⅰ |  |  |  |  |  |  |
| Ⅱ | 1.090 | 0.823-1.443 | 0.549 | 1.07 | 0.807-1.419 | 0.637 |
| Ⅲ | 1.616 | 1.209-2.161 | 0.001 | 1.305 | 0.972-1.751 | 0.076 |
| Ⅳ | 2.061 | 1.465-2.901 | 0.000 | 1.905 | 1.346-2.696 | 0.000 |
| unknown | 2.486 | 1.774-3.482 | 0.000 | 1.509 | 1.064-2.139 | 0.021 |
| N Stage |  |  |  |  |  |  |
| N0 |  |  |  |  |  |  |
| N1 | 0.183 | 0.133-0.252 | 0.000 | 1.587 | 1.396-1.805 | 0.000 |
| N2 | 0.265 | 0.192-0.365 | 0.000 | 2.253 | 1.949-2.605 | 0.000 |
| NX | 0.344 | 0.248-0.477 | 0.000 | 1.928 | 1.362-2.731 | 0.000 |
| Treatment |  |  |  |  |  |  |
| S |  |  |  |  |  |  |
| S+R | 1.021 | 0.590-0.1.767 | 0.941 | 1.22 | 0.703-2.116 | 0.480 |
| S+C | 0.636 | 0.570-0.710 | 0.000 | 0.686 | 0.608-0.774 | 0.000 |
| S+R+C | 0.541 | 0.426-0.686 | 0.000 | 0.666 | 0.520-0.852 | 0.001 |
| CEA |  |  |  |  |  |  |
| normal |  |  |  |  |  |  |
| abnormal | 1.561 | 1.355-1.798 | 0.000 | 1.453 | 1.260-1.677 | 0.000 |
| unknown | 1.441 | 1.222-1.628 | 0.000 | 1.261 | 1.091-1.458 | 0.002 |
| Node removed |  |  |  |  |  |  |
| <12 |  |  |  |  |  |  |
| ≥12 | 0.362 | 0.322-0.407 | 0.000 | 0.367 | 0.323-0.417 | 0.000 |
| unknown | 0.779 | 0.465-1.304 | 0.342 | 0.659 | 0.390-1.116 | 0.121 |
| Size |  |  |  |  |  |  |
| <5 |  |  |  |  |  |  |
| ≥5 | 0.895 | 0.781-1.025 | 0.110 | 0.963 | 0.838-1.107 | 0.599 |
| unknown | 1.999 | 1.633-2.448 | 0.000 | 1.429 | 1.135-1.799 | 0.002 |


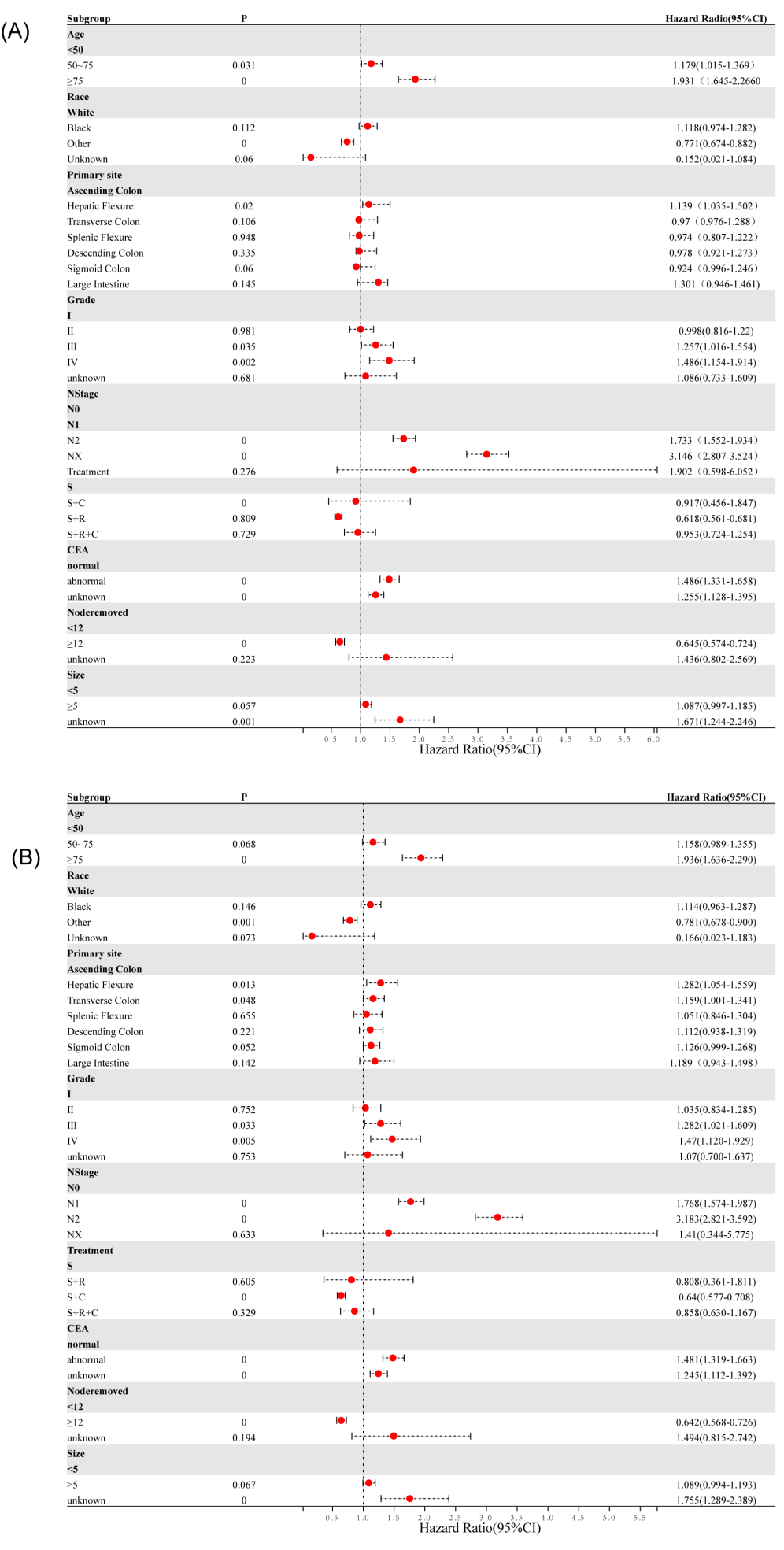


**Figure S1** Forest plots of prognosis analysis of OS and CSS of pT4aM0 COAD. (A) OS of pT4aM0 COAD; (B) CSS of pT4aM0 COAD


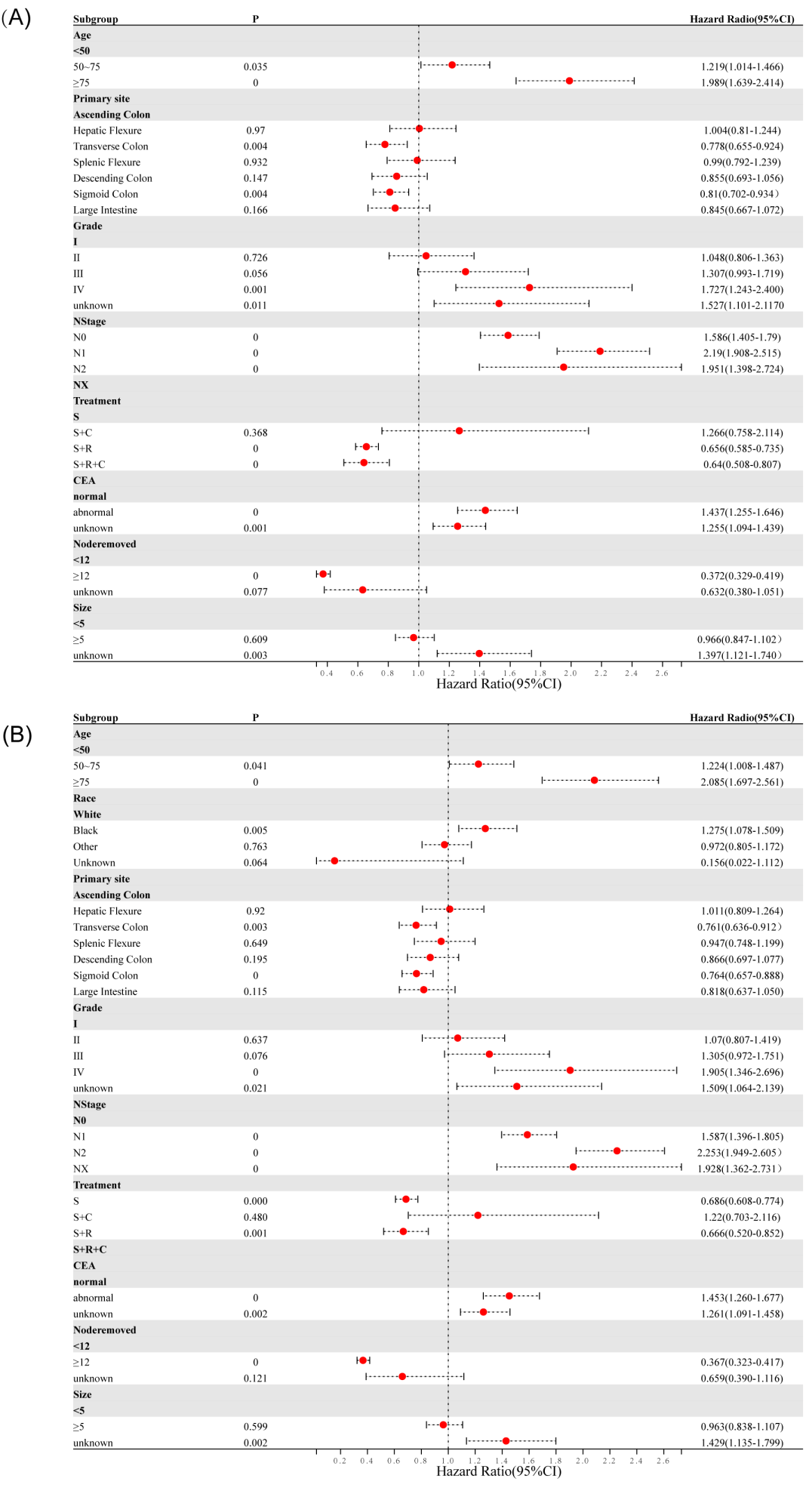


**Figure S2** Forest plots of prognosis analysis of OS and CSS of pT4bM0 COAD. (A) OS of pT4bM0 COAD; (B) CSS of pT4bM0 COAD
